# Lck Mediates Signal Transmission from CD59 to the TCR/CD3 Pathway in Jurkat T Cells

**DOI:** 10.1371/journal.pone.0085934

**Published:** 2014-01-15

**Authors:** Anna M. Lipp, Kata Juhasz, Christian Paar, Christoph Ogris, Paul Eckerstorfer, Roland Thuenauer, Jan Hesse, Benedikt Nimmervoll, Hannes Stockinger, Gerhard J. Schütz, Ulrich Bodenhofer, Zsolt Balogi, Alois Sonnleitner

**Affiliations:** 1 Center for Advanced Bioanalysis GmbH, Linz, Austria; 2 Institute for Bioinformatics, Johannes Kepler University Linz, Linz, Austria; 3 Molecular Immunology Unit, Institute for Hygiene and Applied Immunology, Center for Pathophysiology, Infectiology and Immunology, Medical University Vienna, Vienna, Austria; 4 Institute of Applied Physics, Vienna University of Technology, Vienna, Austria; University of New South Wales, Australia

## Abstract

The glycosylphosphatidylinositol (GPI)-anchored molecule CD59 has been implicated in the modulation of T cell responses, but the underlying molecular mechanism of CD59 influencing T cell signaling remained unclear. Here we analyzed Jurkat T cells stimulated via anti-CD3ε- or anti-CD59-coated surfaces, using time-resolved single-cell Ca^2+^ imaging as a read-out for stimulation. This analysis revealed a heterogeneous Ca^2+^ response of the cell population in a stimulus-dependent manner. Further analysis of T cell receptor (TCR)/CD3 deficient or overexpressing cells showed that CD59-mediated signaling is strongly dependent on TCR/CD3 surface expression. In protein co-patterning and fluorescence recovery after photobleaching experiments no direct physical interaction was observed between CD59 and CD3 at the plasma membrane upon anti-CD59 stimulation. However, siRNA-mediated protein knock-downs of downstream signaling molecules revealed that the Src family kinase Lck and the adaptor molecule linker of activated T cells (LAT) are essential for both signaling pathways. Furthermore, flow cytometry measurements showed that knock-down of Lck accelerates CD3 re-expression at the cell surface after anti-CD59 stimulation similar to what has been observed upon direct TCR/CD3 stimulation. Finally, physically linking Lck to CD3ζ completely abolished CD59-triggered Ca^2+^ signaling, while signaling was still functional upon direct TCR/CD3 stimulation. Altogether, we demonstrate that Lck mediates signal transmission from CD59 to the TCR/CD3 pathway in Jurkat T cells, and propose that CD59 may act via Lck to modulate T cell responses.

## Introduction

Engagement of the TCR/CD3 complex by anti-CD3 antibodies is thought to mimic antigen recognition and initiates protein-tyrosine kinase dependent signaling [1]. A central molecule in this process is the Src family kinase Lck which phosphorylates the immunoreceptor tyrosine-based activation (ITAM) motifs of the TCR/CD3 complex [2]. Phosphorylated ITAMs allow further downstream signaling, leading to changes in intracellular free calcium concentration ([Ca^2+^]_i_) and ultimately altered gene expression critical for T cell activation and survival [3].

In addition to signaling elicited by direct TCR engagement, accessory molecules expressed on the T cell surface play a pivotal role in the modulation of T cell responses. The glycosylphosphatidylinositol (GPI)-anchored molecule CD59 is expressed in almost all cell membranes. It is known to inhibit the complement system by binding to the C8/9 components of the membrane attack complex, thereby preventing its assembly and formation of the lytic pore [4]. Besides its role as a complement system inhibitor, signaling capacity of CD59 in T cells has been demonstrated. Although TCR/CD3- and CD59-mediated signaling pathways are considered clearly distinguishable with respect to the membrane localization of the TCR/CD3 complex and CD59 [5]–[7], there have been implications for a potential overlap of the elicited signaling pathways [8], [9]. Antibody (Ab)-mediated cross-linking of CD59 on human T cells has been shown to trigger signaling events similar to those observed upon TCR triggering. These include phosphorylation of protein tyrosine kinases, elevation of [Ca^2+^]_i_, as well as proliferation and interleukin (IL)-2 production upon phorbol-12-myristate-13-acetate (PMA) co-stimulation [8]–[10]. Intriguingly, some of these events were dependent on TCR/CD3 co-expression, while others were found independent [8]. Whereas previous data indicated a positive regulatory role, recent studies showed a possible negative regulatory role of CD59 in T cell activation, as both blockade and siRNA-mediated knock-down of CD59 caused enhanced antigen-specific responses in human T cells [11], [12]. Interestingly, it has been shown that knock-down of CD59 or CD59-deficiency affected the T cell response only in the presence of potential ligands such as antigen presenting cells (APCs) or antibodies [12], [13].

Although accumulating data suggest a physiological role for CD59 in T cell activation, the mechanism how signaling via CD59 is transduced through the membrane to modulate the antigen-specific T cell response remains to be explored. In this study we addressed whether and how CD59-mediated signaling is coupled to the TCR/CD3-mediated signaling cascade, using Jurkat cells as a model system. A rise in [Ca^2+^]_i_ is one of the earliest events upon T cell activation and different types of Ca^2+^ responses are critical for the differential activation of transcription factors driving T cell proliferation and effector functions [14]–[16]. Here we used single-cell Ca^2+^ measurements of Jurkat T cells as a read-out for signaling elicited upon Ab-mediated cross-linking of CD59 vs. TCR/CD3 stimulation. It is shown with a series of mutants and siRNA-mediated protein knock-downs that Lck is a key component in coupling CD59-mediated signaling to the TCR/CD3-mediated signaling pathway in Jurkat T cells.

## Materials and Methods

### Antibodies and reagents

CD3 MEM-57 (Abcam), CD59 MEM-43, and CD71 monoclonal antibodies (mAb), or appropriate isotype control IgG2a (all AbD Serotec) were used for cell stimulation. For Western blotting β-actin (C4) mAb, CD3-zeta mAb (F-3), LAT mAb (4i355), Lck mAb (3A5) (all SantaCruz Biotechnology), CD3ε pAb (Sigma-Aldrich), CD59 pAb (both Abcam), Fyn mAb (FYN-01) (Exbio Praha), and HRP-labeled goat anti-mouse (Sigma-Aldrich) or donkey anti-rabbit IgG (GE Healthcare) were used. Fluorophore-conjugated antibodies CD3-FITC MEM-57, CD59-FITC MEM-43, and IgG2a-FITC were purchased from AbD Serotec, CD8a-FITC OKT-8 from eBioscience^®^, CD3-AF-647 MEM-57, and IgG2a-AF647 were from Exbio Praha. For inhibition of Src family kinases, cells were incubated with 10 µM PP2 (Molecular Probes) for 30 min at room temperature.

### siRNA and plasmid constructs

Double-stranded 21-mer siRNA (Hs_Lck_3; target sequence: AAGGGCCAGGACTTTATCTAA; Hs_Fyn_5_HP validated siRNA; Hs_LAT_1; target sequence: AAGGGCCAGGACTTTATCTAA) and Allstars validated negative control siRNA were purchased from QIAGEN GmbH. pEFBOS-CD8-ζ [17] was kindly provided by A. Weiss (University of California, San Francisco, CA). The pEYFP-N1 backbone (Clontech Laboratories) was used to construct human CD3ζ-EYFP fusion protein. The hCD3Δcyt-wtLck-mEGFP chimera was constructed by fusing extracellular and transmembrane domain human of CD3ζ with wild-type Lck molecule C-terminally tagged with mEGFP. For retroviral infection of J.CaM1.6 cells, hCD3Δcyt-wtLck-mEGFP was cloned into the retroviral vector pBMN-Z (provided by G. Nolan, Stanford University School of Medicine, Stanford, CA).

### Cells and transfections

Jurkat E6.1 TIB-152 (ATCC) and its derivative mutant cell lines Lck-deficient J.CaM1.6 (European Collection of Cell Cultures, Salisbury, UK), LAT-deficient J.CaM2.5 [18] (kindly provided by A. Weiss), and TCR-negative J31.13 [19] (kindly provided by O. Acuto, Sir William Dunn School of Pathology, Oxford, UK) were maintained in RPMI 1640 supplemented with 10% FBS, 2 mM L-glutamine, 100 units/ml penicillin, and 100 mg/ml streptomycin (all from Invitrogen Life Technologies) at 37 °C and 5% CO_2_. Jurkat cells were transiently transfected with 2.5 µg (200 nM) siRNA, or 1 µg of respective plasmid DNA using Amaxa nucleofection (Lonza Group Ltd) according to the manufacturer's protocol. Cells transfected with CD3ζ-EYFP were selected for positive cells in complete medium supplemented with 1 mg/ml G418 (PAA Laboratories). J.CaM1.6 cells were retrovirally transduced with hCD3Δcyt-wtLck-mEGFP construct using standard molecular biology methods. mEGFP- and EYFP-positive cells were enriched using a FACSAria™ (BD Biosciences) cell sorter.

### Stimulatory surfaces for live cell imaging

Silicone isolators (Sigma-Aldrich) were mounted onto epoxy modified no. 1 glass slides (150 μm, Karl Hecht KG), homogeneously coated with protein A (50 µg/ml; Sigma-Aldrich) followed by incubation with 5 µg/ml stimulatory mAb. Unspecific binding sites were saturated using 2% bovine serum albumin (BSA) (PAA Laboratories).

### Single-cell Ca^2+^ imaging

All live-cell experiments were performed at 37 °C adjusted by an objective heating system (PeCon/Erbach). Fluorescent measurements were performed as previously described [20]. In short, cells were loaded with 5 μM Indo-1/AM (Molecular Probes®) in complete RPMI 1640 at room temperature for 15 min, washed with HBSS (Invitrogen) and flushed onto stimulatory surfaces on the stage of a Zeiss Axiovert microscope. Cells were illuminated using a mercury lamp (HBO100, Zeiss) at 333 (HQ333/30X) nm, and fluorescence emissions collected via a 40x Neofluar objective (Zeiss). The fluorescence light was split into two emission channels using a dichroic beamsplitter (365DCLP) and two bandpass filters at 405 nm (D405/30M) and 485 nm (D485/25M) and detected simultaneously using two charge-coupled device (CCD) cameras (Photometrics). All bandpass filters and dichroic beamsplitter were from AHF Analysentechnik. Movies were acquired at a frame rate of 0.5 frames per second, the emission ratio of 405/485 nm was determined after background correction and preprocessed in MATLAB (MathWorks™ Inc.). Individual cells were selected manually and Ca^2+^ time traces were calculated after background correction. Full Ca^2+^ time traces were processed by first automatically identifying the time of cell-surface contact for each individual cell and second extracting 100 out of 240 total frames to ensure comparable observation windows and equal length of time traces. For noise reduction and elimination of outliers, a median filter with a window size of 10 time points was applied.

### Cluster analysis of Ca^2+^ time traces

For clustering of Ca^2+^ time traces, affinity propagation clustering was used [21]. Affinity propagation has two major advantages: (i) it allows identifying most typical representatives for each cluster, the so-called exemplars, and (ii) it does not require any particular properties of the similarity measure that is used for clustering. We tailored the similarity measure to the special needs of clustering Ca^2+^ time traces: we used negative squared distances, since both absolute signal intensities and exact time points are to be considered, but admitted for small shifts to account for the fact that starting points of signals cannot be determined exactly. More specifically, for any two signals x =  (x_1_,..,x_i_) and y =  (y_1_,..,y_i_), we compute their similarity as

s (x,y)  = – min _k  =  −10,…,10_ Σ(x_i_ – y_i-k_)^2^.

In addition, specification of an input preference parameter which controls the granularity of the result is required. Based on empirical evaluations an input preference corresponding to a linear 2:98 split between the minimal and maximal input preference (as determined by Frey's and Dueck's preference range algorithm [21]) was applied. The clustering procedure has been implemented in the R statistical computing platform making use of a recent R implementation of affinity propagation [22]. Ca^2+^ time traces for each figure were clustered together resulting in 10–12 clusters for each figure. Percentages of Ca^2+^ time traces for each cluster were calculated for each experiment independently, and mean values ± SD were calculated.

### Ab-micropatterned surfaces and total internal reflection fluorescence (TIRF) microscopy

Micropatterned glass surfaces were prepared as described earlier [23] with only slight modifications. In short, polydimethylsiloxane stamps were incubated with 100 mg/ml Cy-5 labeled BSA (labeled via Cy-5 Mono 5 Pack for Labeling, GE Healthcare) for 30 min at room temperature and placed for 30 min on epoxy modified glass slides. Upon removal of the stamps, the patterned area was confined by silicone isolators (Sigma-Aldrich) and stimulatory surfaces were prepared as described above. BSA efficiently blocked unspecific adsorption of both protein A and mAb, thereby providing a well-defined 3 μm micropattern. Fluorescence of EYFP or Cy5 was excited by a Kr^+^/Ar^+^ mixed gas laser (Innova, Coherent) at 514 nm or 647 nm, respectively. Samples were illuminated in TIRF configuration using a 100x, N.A.  = 1.46 alpha Plan-Apochromat objective (Zeiss) and a TIRF condenser (Till-Photonics). After appropriate filtering using standard filter sets (bandpass HQ575/90M, dichroic beamsplitter Z514/780 for EYFP; bandpass HQ700/75M, dichroic beamsplitter Q660LP for Cy5) (AHF Analysentechnik), fluorescence was imaged onto a CCD camera (Photometrics). Images were acquired sequentially in two colors, with one at 514 nm (EYFP) and one at 647 nm (Cy5) using 150 ms exposure time and 3x binning, resulting in a pixel size of 190 nm for all images. Filters and beamsplitters were exchanged between the images.

### Fluorescence recovery after photobleaching (FRAP) measurements

FRAP experiments were performed on a Zeiss Axiovert microscope equipped with a temperature control (POCmini; Zeiss) and custom-built incubation box. EYFP in samples was illuminated through a 100x, NA =  1.45 alpha Plan-Apochromat objective (Zeiss) using the 514 nm line of an Ar^+^-ion laser (Innova, Coherent). A slit aperture (Zeiss) was used as field stop to confine an illumination area of 5 µm x 7 µm. After appropriate filtering (custom-made dichroic beamsplitters and emission filters, Chroma Technology Corp.), images were recorded on a CCD camera (Micro Max 1300-PB, Roper Scientific). For the precise control of all laser pulse trains, an acusto-optical modulator (1205C; Isomet) was used. Three pre-bleach images were recorded with attenuated laser power, an illumination time of 1 ms, and a delay of 10 s between subsequent images followed by a photo-bleaching laser pulse applied for 40 ms with high laser power. After a recovery time of 1000 ms, a sequence of recovery images (1 ms illumination time, 10 s delay) was recorded at low laser power. Timing protocols were generated by a program package implemented in LABVIEW (National Instruments). FRAP data were analyzed by MATLAB and normalized by the first pre-bleach image. For determination of the halftime of recovery (t_half_) the function: y(t)  = A*(1-exp(– τ * t)) was fitted to all data sets; A accounts for the final recovered intensity, τ is the fitted parameter and t is the time after the bleaching pulse. After determination of τ by fitting the above equation to the recovery curve the corresponding halftime of the recovery can be calculated: t_half_ =  ln(0.5)/ – τ.

### Cell stimulation and surface labeling

For Ab-induced cross-linking in solution, Jurkat cells were primed with 10 µg/ml CD59 mAb MEM-43 or IgG2a (both AbD Serotec) for 5 min followed by incubation with 80 µg/ml cross-linking F(ab)_2_ fragments of goat anti-mouse IgG (Sigma-Aldrich) at 37 °C in complete medium for the times indicated. Stimulation was stopped by adding ice-cold staining buffer (1x PBS supplemented with 2% FCS, 1 mM CaCl_2_, and 0.5 mM MgCl_2_). For surface staining 0.25 µg of fluorophore-conjugated mAbs were added to 5×10^5^ cells. Cells were incubated for 30 min at 4 °C, excessively washed, and resuspended in 250 µl ice-cold staining buffer. For exclusion of damaged cells propidium-iodide (4 µg/ml, Sigma-Aldrich) was added. Using flow cytometry (BD FACSAria™), FITC or AF647 fluorescence in samples was excited at 488 nm or 633 nm, respectively, and detected using a bandpass filter of 530/30 nm or 660/20 nm, respectively. Cells were gated by Forward Scatter vs. Side Scatter plotting and propidium-iodide exclusion (bandpass filter of 695/40 nm). Mean fluorescence values of 10,000 cells were calculated and corrected with isotype controls.

### SDS-PAGE and Western blot analysis

For Western blot analysis cells were washed with ice-cold PBS and lysed for 30 min in ice-cold lysis buffer (50 mM Tris-HCl (pH 7.5), 5 mM EDTA, 150 mM NaCl) containing 1% Triton X-100 and protease inhibitors (leupeptin (4 µg/ml), aprotinin (4 µg/ml), and PMSF (500 µM) under reducing conditions (2 mM 2-ME) (all from Sigma-Aldrich). After centrifugation (for 5 min at 14,000 x g at 4 °C) equal amounts of cell lysate were separated on 12% SDS gels and transferred to PVDF membranes (Immobilon™-P; Millipore Corporation). Membranes were probed with protein-specific Abs and HRP-conjugated secondary Abs. Signals were visualized by ECL reagent (GE Healthcare) using the Fusion SL image acquisition system (Vilber Lourmat).

### Ensemble Ca^2+^ measurements

Prior to stimulation in solution, cells were labeled with 5 µM Indo-1/AM and ensemble Ca^2+^ measurements were performed using a fluorescence microplate reader (Infinite M200Pro, Tecan Group Ltd.) (excitation 340 nm, emission 410/480 nm) in 96-well UV-transparent microplates (Greiner Bio-One). For all measurements, the bandwidths of excitation and emission were 20 nm and 9 nm, respectively; the integration time was 2000 µs; the gain was 140. Background fluorescence was subtracted to compensate for variations in dye loading.

### Statistical analysis

Statistical analysis was performed using SigmaPlot. All values are expressed as mean ±SD and statistical significance for differences in independent data sets was calculated using one-way ANOVA (Holm-Sidak multiple comparison test). * p < 0.05, ** p < 0.01, *** p < 0.001.

## Results

### Jurkat cells show heterogeneous Ca^2+^ signaling upon anti-CD3 and anti-CD59 stimulation

It has been previously reported that Ab-mediated cross-linking of CD59 results in elevation of [Ca^2+^]_i_ in Jurkat cells [5], [9], [24]. Elevation of [Ca^2+^]_i_ is an early read-out for T cell activation, where type and extent of the Ca^2+^ response are indicative of stimulation [25]. To investigate stimulation via Ab-mediated cross-linking of CD59 vs. TCR/CD3, we performed single-cell based Ca^2+^ measurements of Jurkat cells. Cells were stimulated via Ab-coated glass surfaces, an established approach for studying T cell activation [26], using either anti-CD59 or anti-CD3ε mAbs (referred to as anti-CD59 or anti-CD3 stimulation, respectively). Changes in [Ca^2+^]_i_ during stimulation were recorded for 200 s after initial contact of the individual cell with the stimulatory surface, imaging the ratiometric fluorescent Ca^2+^ indicator Indo-1/AM [27] (Figure S1). In this way changes in [Ca^2+^]_i_ were monitored over time at the single-cell level. For a simplified and unsupervised analysis we implemented cluster analysis of individual Ca^2+^ time traces. The cluster algorithm is based on affinity propagation [21], ensuring distinct, yet homogeneous clusters of Ca^2+^ time traces (see Materials and Methods).

**Figure 1:**
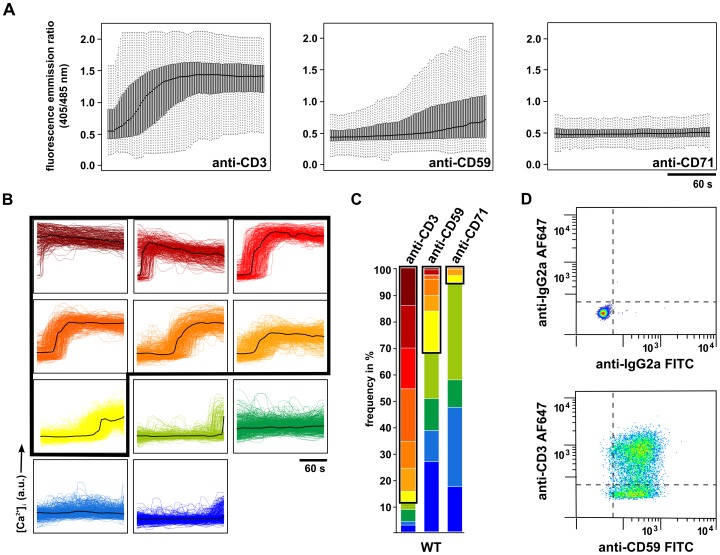
Single-cell Ca^2+^ measurements reveal differential heterogeneity upon anti-CD3 and anti-CD59 stimulation. Jurkat cells (WT) were loaded with Indo-1/AM followed by stimulation with anti-CD3-, anti-CD59-, or anti-CD71-coated surfaces. Individual Ca^2+^ time traces were measured for 200 s after identification of initial cell-surface contact as described in Figure 1S. (A) Box plots of individual Ca^2+^ time traces generated by analysis of the whole cell population upon anti-CD3, anti-CD59, and anti-CD71 stimulation (black line  =  median, grey area  = 50% of Ca^2+^ time traces, dotted lines  =  75% of Ca^2+^ time traces). (B) Individual Ca^2+^ time traces from single-cell measurements were grouped into 11 clusters by affinity propagation clustering as described in Materials and Methods. Each plot shows the respective Ca^2+^ time traces for a cluster, an exemplar trace for each cluster is shown in black. Clusters representing Ca^2+^ release patterns are framed in black. (C) Stimulus-dependent cluster distribution upon anti-CD3, anti-CD59, and anti-CD71 stimulation in WT cells is shown by stacked bar plots. Each color represents the percentage of a certain Ca^2+^ time trace cluster in the cell population. Clusters representing Ca^2+^ release patterns are framed in black (88.5±4.9%, 31.8±12.0%, and 5.5±5.3% for anti-CD3, anti-CD59, and anti-CD71 stimulation, respectively). Mean values from at least three independent experiments, each with three technical replicates, are shown (n ≥ 249 per stimulatory condition). (D) CD3ε and CD59 surface expression levels in WT cells. WT cells were surface stained with Alexa Fluor 647-conjugated anti-CD3ε and FITC-conjugated anti-CD59 or isotype controls and analyzed by flow cytometry. Live cells were gated based on the Forward Scatter and Side Scatter profiles and propidium iodide exclusion. A representative dot plot of four technical replicates is shown.

Consistent with previous reports [9], [24], on average the Jurkat population data revealed a higher stimulatory potential for anti-CD3 compared to anti-CD59 stimulation. Upon anti-CD3 stimulation there was a rapid rise in [Ca^2+^]_i_ followed by a sustained elevation of [Ca^2+^]_i_, whereas there was a less pronounced increase resulting in lower levels of total [Ca^2+^]_i_ upon anti-CD59 stimulation (Figure 1*A*). Surfaces coated with mAb against the transferrin receptor (CD71) as a control triggered no considerable increase in [Ca^2+^]_i_ (Figure 1*A*). When applying the cluster analysis to the data obtained by anti-CD3 and anti-CD59 stimulation of wild-type (WT) Jurkat cells, both types of stimulation displayed heterogeneity in Ca^2+^ signaling. The individual Ca^2+^ time traces generated by the Jurkat population were clustered into groups of similar traces, differing in amplitude, on-set time, and the dynamic pattern of [Ca^2+^]_i_ (Figure 1*B*). In Figure 1*B* Ca^2+^ time traces for each cluster are shown. Ca^2+^ time traces displayed in red to yellow (Figure 1*B*, framed) exhibit a sharp increase followed by sustained elevation of [Ca^2+^]_i_, known to be critical for activation, and are hereinafter referred to as Ca^2+^ release patterns. Ca^2+^ time traces hereinafter referred to as cells not showing Ca^2+^ release patterns include those showing transient spikes of elevated [Ca^2+^]_i_ and Ca^2+^ time traces which practically do not show changes in [Ca^2+^]_i_ (Figure 1*B*, green to blue). Although all clusters of Ca^2+^ time traces appeared for both types of stimulation (Figure 1*C*), the cluster distribution of Ca^2+^ time traces in the Jurkat population differed greatly for anti-CD3 and anti-CD59 stimulation. The number of cells showing Ca^2+^ release patterns was less upon anti-CD59 stimulation compared to anti-CD3 stimulation (31.8±12.0% vs. 88.5±4.9%, respectively) (Figure 1*C*, framed). It is noted that on non-activating anti-CD71-coated surfaces only 5.5±5.3% of the Jurkat population showed Ca^2+^ release patterns (Figure 1*C*, framed).

Since all experiments were performed at maximum signaling capacity for the stimuli (for titration of Ab concentration on the glass substrate see Figure S2 *A*, S2*B*), the observed difference in cluster distribution for anti-CD3 and anti-CD59 stimulation was not due to unsaturated stimulation conditions. Note that using an alternative mAb to CD59 (clone H19) yielded similar results (data not shown). Besides a possible dependence of cluster distribution on stimulus strength, the signaling capacity of a single cell may also be determined by the amount of the targeted surface molecules. Measuring CD59 surface expression by flow cytometry was found homogeneous throughout the Jurkat population. By contrast, CD3 surface expression was heterogeneous in the Jurkat population, uncovering a significant proportion of cells displaying undetectable CD3 surface expression levels (Figure 1*D* *)*. This finding raised the possibility that the heterogeneity in CD59-mediated Ca^2+^ signaling may be linked to the heterogeneity in CD3 surface expression levels in Jurkat cells.

### CD3ζ surface expression is essential for CD59-mediated Ca^2+^ signaling

**Figure 2:**
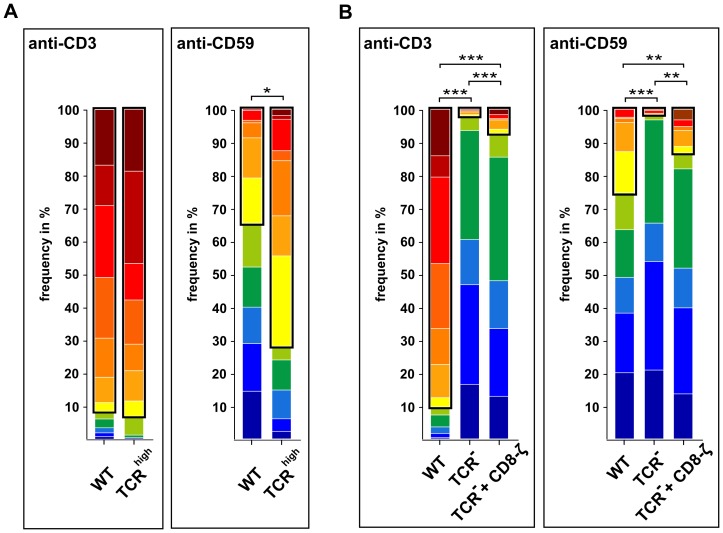
CD59-mediated Ca^2+^ signaling requires CD3ζ expression. (A) Cluster distribution of Ca^2+^ time traces in WT and TCR^high^ cells is shown upon anti-CD3 and anti-CD59 stimulation. Each color represents the percentage of a certain Ca^2+^ time trace cluster in the cell population. Clusters representing Ca^2+^ release patterns are framed in black (91.4±2.1% and 92.9±3.9% upon anti-CD3 stimulation, 34.4±16.3% and 72.0±16.6% upon anti-CD59 stimulation in WT and TCR^high^ cells, respectively). Mean values from two independent experiments, each with three technical replicates are shown (n ≥ 167 per cell type and condition). (B) Cluster distribution of Ca^2+^ time traces in WT, TCR^-^, and cells expressing CD8-ζ fusion protein is shown upon anti-CD3 and anti-CD59 stimulation. Each color represents the percentage of a certain Ca^2+^ time trace cluster in the cell population. Clusters representing Ca^2+^ release patterns are framed in black (90.1±1.4%, 1.9±0.7% and 7.4±2.3% upon anti-CD3 stimulation, 25.2±6.8%, 1.6±0.4% and 13.4±1.6% upon anti-CD59 stimulation for WT, TCR^-^, and CD8-ζ cells, respectively). Mean values from four independent experiments, each with three technical replicates, are shown (n ≥ 343 per cell type and condition). Multiple comparison tests for the fractions showing Ca^2+^ release patterns in (A) and (B) were assessed by one-way ANOVA, significances are shown where applicable, * p < 0.05, ** p < 0.01, *** p < 0.001.

To test the idea of CD59-mediated signaling being dependent on CD3 expression levels, we examined Ca^2+^ signaling in response to anti-CD59 stimulation in Jurkat cells overexpressing the TCR/CD3 complex, generated by stable transfection with EYFP-tagged CD3ζ (TCR^high^). The Ca^2+^ signaling capacity in TCR^high^ cells compared to WT Jurkat cells was measured via single-cell Ca^2+^ measurements for both types of stimulation (Figure 2*A*, for clustering of single-cell Ca^2+^ time traces see Figure S3 *A*, for TCR/CD3 surface expression levels see S3*B*, for total CD3ζ expression levels see S3*C*). Upon anti-CD3 stimulation, there was no change in the number of cells showing Ca^2+^ release patterns in TCR^high^ cells compared to WT cells (92.9±3.9% vs. 91.4±2.1%) (Figure 2*A*, framed). By contrast, there was a significant increase in the number of Ca^2+^ release patterns upon anti-CD59 stimulation, values rising from 34.4±16.3% in WT cells to 72.0±16.6% in TCR^high^ cells (Figure 2*A*, framed). It is noted that CD59 expression levels were not altered in TCR^high^ cells as compared to their WT counterparts (Figure S3 *D*).

Since increased CD3 expression levels gave rise to enhanced Ca^2+^ signaling upon anti-CD59 stimulation, we examined if Ca^2+^ signaling upon anti-CD59 stimulation could be elicited in a Jurkat mutant lacking the TCR/CD3 complex on the cell surface (TCR^-^) (for TCR/CD3 surface expression levels see Figure S3 *B*). Ca^2+^ signaling was essentially abolished in TCR^-^ cells upon anti-CD59 stimulation (1.6±0.4% Ca^2+^ release patterns), as well as for anti-CD3 stimulation used as a control (1.9±0.7% Ca^2+^ release patterns) (Figure 2*B*, framed). It is noted that the store-operated Ca^2+^ entry mechanism was fully functional in TCR^-^ cells as tested by adding 2 µM of the sarco/endoplasmic reticulum Ca^2+^-ATPase pump inhibitor thapsigargin (data not shown) and CD59 expression levels were similar in WT and TCR^-^ cells (Figure S3 *D*). Moreover, when reconstituting ITAM domains in TCR^-^ cells via introducing a construct where the cytoplasmic tail of CD3ζ is fused to CD8 (CD8-ζ), Ca^2+^ signaling upon anti-CD59 stimulation could be partially restored. The number of cells showing Ca^2+^ release patterns upon anti-CD59 stimulation raised from 1.6±0.4% in TCR^-^ cells to 13.4±1.6% in CD8-ζ cells, thereby reconstituting 53% of the Ca^2+^ release patterns observed in WT cells (25.2±6.8%). Upon anti-CD3 stimulation Ca^2+^ signaling in CD8-ζ cells was scarcely restored (7.4±2.3% Ca^2+^ release patterns) compared to WT cells (90.1±1.4% Ca^2+^ release patterns) (Figure 2*B*, framed). The partial reconstitution upon anti-CD59 stimulation can be explained by the varying transfection efficiency of 32.4±10.2% CD8-positive cells as tested via flow cytometry (Figure S3 *E*). Altogether, these results demonstrated a critical role of TCR/CD3 for both stimuli. Furthermore, we could show that CD3ζ even without the ligand-binding components of the TCR/CD3 complex is utilized for CD59-mediated signaling pointing to CD59-mediated signaling being linked to the TCR/CD3-mediated route.

### Physical interaction between CD59 and CD3 at the plasma membrane was not visible upon anti-CD59 stimulation

**Figure 3:**
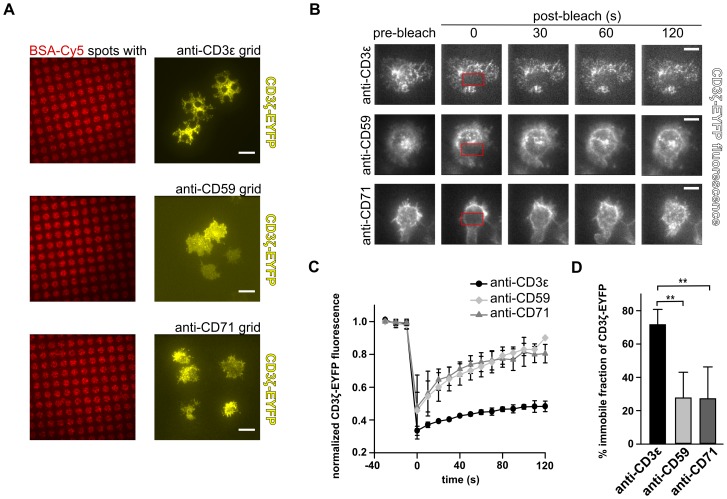
Neither static nor dynamic interaction between CD59 and CD3 at the cell surface is visible upon anti-CD59 stimulation. (A) Distribution of CD3ζ-EYFP in Jurkat cells on Ab-patterned surfaces. Non-Ab-coated BSA-Cy5 spots are shown as a control in the left column. In the right column, TIRF microscopy images of Jurkat cells expressing CD3ζ-EYFP are shown on (a) anti-CD3ε, (b) anti-CD59, or (c) anti-CD71 grids (scale bars  =  10 µm). (B) FRAP measurements of CD3ζ-EYFP in Jurkat cells. After acquiring pre-bleach images, CD3ζ-EYFP was bleached in a defined area (red rectangle) at the bottom of the cell attached to anti-CD3ε-, anti-CD59-, or anti-CD71-coated surfaces. Recovering fluorescence intensity in the area was imaged at indicated time points (scale bars  =  5 µm). (C) Fluorescence recovery curves of CD3ζ-EYFP in cells plated on anti-CD3ε-, anti-CD59-, or anti-CD71-coated surfaces (mean ±SD). (D) Immobile fractions of CD3ζ-EYFP calculated from fluorescence recovery curves. Representative data of two separate experiments are shown (mean ±SD, n≥3). Multiple comparison tests were assessed by one-way ANOVA, significances are shown where applicable, ** p<0.01.

To unravel the mechanism how the CD59-mediated signaling pathway integrates into the TCR/CD3-mediated signaling pathway, we tested if CD59 and CD3 are physically associated at the plasma membrane upon anti-CD59 stimulation. For this, a live cell protein-protein interaction assay was used that is based on co-patterning of a fluorescent target molecule in the cell with a membrane protein immobilized via capture Abs micropatterned on surfaces. In case of protein-protein interaction, the fluorescent target molecule follows the imposed micropattern in the membrane [23]. This approach was implemented using Jurkat cells expressing CD3ζ-EYFP as a target molecule. After plating cells on anti-CD3ε-, anti-CD59, or anti-CD71-patterned surfaces, localization of CD3ζ-EYFP was visualized via TIRF microscopy, imaging only the close proximity of the cellular membrane. Stimulation of cells via anti-CD3ε-grid surfaces, complement to the BSA-Cy5-coated spots, resulted in redistribution of CD3ζ-EYFP in the plasma membrane along with the imposed CD3ε pattern (Figure 3*A*, top panel). These results indicated an interaction between CD3ε and CD3ζ-EYFP, which served as a positive control. When testing anti-CD59-patterned surfaces, CD3ζ-EYFP remained uniformly distributed at the cellular surface (Figure 3*A*, middle panel). Similarly, CD3ζ-EYFP remained uniformly distributed in cells exposed to anti-CD71-patterned surfaces (Figure 3*A*, bottom panel). Note that anti-CD71-patterned surfaces did not stimulate significant Ca^2+^ signaling and served as a negative control. These data revealed no apparent interaction between CD3 and CD59 upon anti-CD59 stimulation on a minute timescale.

Further focusing on the plasma membrane, a possible, more dynamic interaction between CD3 and CD59 upon anti-CD59 stimulation was tested by FRAP measurements with CD3ζ-EYFP expressing Jurkat cells. Cells were exposed to surfaces uniformly coated with anti-CD3ε, anti-CD59 or anti-CD71 mAbs. After bleaching EYFP-fluorescence in a defined region of the plasma membrane, recovery of fluorescence intensity was followed for 2 min (Figure 3*B*). As expected, fluorescence recovery of CD3ζ-EYFP was slow on anti-CD3ε-coated surfaces, indicating a strong immobilization of the molecule by the anti-CD3ε-coated surface (Figure 3*C*). By contrast, fluorescence recovery of CD3ζ-EYFP remained fast on anti-CD59-coated surfaces, comparable to that measured on anti-CD71-coated surfaces (Figure 3*C*). Furthermore, CD3ζ-EYFP displayed an expected large immobile fraction on anti-CD3ε-coated surfaces (71.5±9.2%). Meanwhile, on both anti-CD59- and anti-CD71-coated surfaces mainly mobile CD3ζ-EYFP molecules were observed, with comparable low immobile fractions of 27.6±15.4% and 27.1±19.1%, respectively (Figure 3*D*). These results showed no apparent co-immobilization of CD3ζ-EYFP with immobilized CD59 or CD71 at the plasma membrane. Taken together, our live cell imaging data revealed no visible physical interaction at the plasma membrane between CD3 and CD59 upon anti-CD59 stimulation at a second to minute timescale.

### Lck and LAT, but not Fyn are critical for CD59- and TCR/CD3-mediated Ca^2+^ signaling

Since we could not identify a direct physical interaction between CD3 and CD59 at the plasma membrane, we tested downstream molecules involved in TCR/CD3-mediated signaling as candidates for coupling the two pathways. Molecules tested were the Src family kinases, Lck and Fyn, as the ITAM domains residing on CD3ζ act as a major target for these molecules [28]–[30]. We also tested LAT, a central adaptor molecule in TCR/CD3-mediated signaling [31].

**Figure 4:**
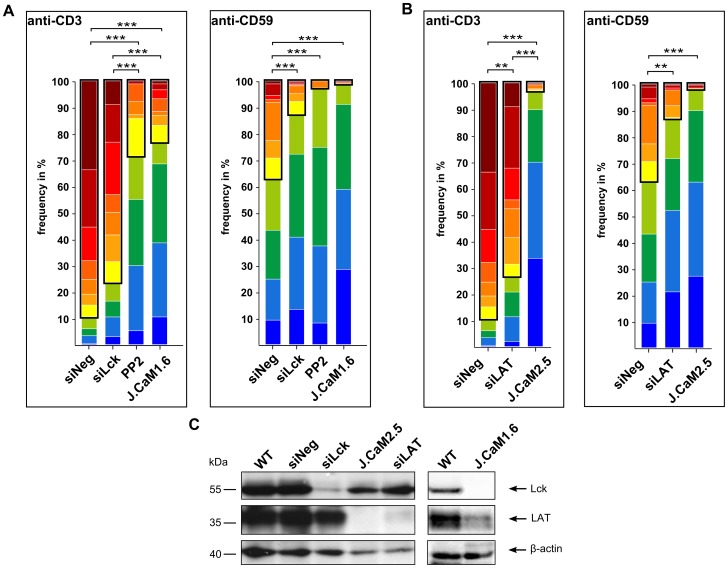
TCR/CD3- and CD59-mediated Ca^2+^ signaling are dependent on Lck and LAT. Cluster distribution of Ca^2+^ time traces in differently treated cells upon anti-CD3 and anti-CD59 stimulation. Each color represents the percentage of a certain Ca^2+^ time trace cluster in the cell population. (A) Ca^2+^ measurements were performed with WT cells transiently transfected with negative control siRNA (siNeg), Lck-specific siRNA (siLck), WT cells treated with 10 µM PP2 (PP2), and Lck-deficient J.CaM1.6 cells. Clusters representing Ca^2+^ release patterns are framed in black (89.4±10.1%, 76.5±10.4%, 28.3±39.1%, and 23.4±9.9% upon anti-CD3 stimulation, 36.9±13.2%, 12.7±6.1%, 2.6±2.0%, and 1.2±1.2% upon anti-CD59 stimulation for siNeg, siLck, PP2-treated, and J.CaM1.6 cells, respectively). Mean values from at least two independent experiments, each with three technical replicates, are shown (n ≥ 204 per cell type and condition). (B) Cluster analysis of Ca^2+^ time traces in WT cells transiently transfected with negative control siRNA (siNeg), LAT-specific siRNA (siLAT), and LAT-deficient J.CaM2.5 cells. Clusters representing Ca^2+^ release patterns are framed in black (89.4±10.1%, 73.5±5.8%, and 3.0±2.3% upon anti-CD3 stimulation, 36.9±13.2%, 13.0±6.6%, and 1.7±1.6% upon anti-CD59 stimulation for siNeg, siLAT, and J.CaM2.5 cells, respectively). Mean values from at least three independent experiments, each with three technical replicates, are shown (n ≥ 208 per cell type and condition). Multiple comparison tests for the fractions showing Ca^2+^ release patterns in (A) and (B) were assessed by one-way ANOVA, significances are shown where applicable, ** p < 0.01, *** p < 0.001. (C) Knock-down of target proteins was tested by Western blotting. 48 h after transfection cell lysates from siRNA treated cells were probed with anti-Lck, anti-LAT, and anti-β-actin. J.CaM1.6 cells, J.CaM2.5 cells, and WT cells served as controls.

When applying our single-cell Ca^2+^ technique to siRNA-mediated Lck knock-down cells (siLck), there was no significant difference in the number of cells showing Ca^2+^ release patterns upon anti-CD3 stimulation compared to cells transfected with negative control siRNA (siNeg) (76.5±10.4% vs. 89.4±10.1%). By contrast, the number of cells showing Ca^2+^ release patterns did significantly decrease from 36.9±13.2% to 12.7±6.1% when comparing siNeg to siLck cells upon anti-CD59 stimulation (Figure 4*A*, framed). For clustering of single-cell Ca^2+^ time traces see Figure S4 *A,* for knock-down efficiency see Figure 4*C*. Upon anti-CD3 stimulation, treatment of WT cells with the Src family kinase inhibitor PP2 significantly decreased the number of cells showing Ca^2+^ release patterns compared to siNeg cells (28.3±39.1% vs. 89.4±10.1%, respectively) (Figure 4*A*, framed). Upon anti-CD59 stimulation Ca^2+^ signaling was essentially abolished in PP2-treated cells (2.6±2.0% Ca^2+^ release patterns) (Figure 4*A*, framed). It is noted that siNeg cells were not different from the untreated WT cells in terms of Ca^2+^ signaling (see Figure 1*C*). Thus, Lck proved to be a critical molecule for both CD59- and TCR/CD3-mediated Ca^2+^ signaling, which was supported by single-cell Ca^2+^ measurements with the Lck-deficient Jurkat cell line (J.CaM1.6). This cell line exhibited significantly less Ca^2+^ release patterns compared to siNeg cells upon anti-CD3 stimulation (23.4±9.9% vs. 89.4±10.1%, respectively) and showed hardly any Ca^2+^ signaling upon anti-CD59 stimulation (1.2±1.2% Ca^2+^ release patterns) (Figure 4*A*, framed). It is noted that CD3 and CD59 expression levels in J.CaM1.6 cells were similar to that of WT cells (Figure S4 *B*).

siRNA-mediated knock-down of Fyn (siFyn) (for efficiency of knock-down see Figure S4 *C*) did not significantly affect Ca^2+^ signaling upon anti-CD3 stimulation (79.6±11.0% vs. 89.4±10.1% Ca^2+^ release patterns for siFyn vs. siNeg, respectively). Furthermore, upon anti-CD59 stimulation the number of cells showing Ca^2+^ release patterns was comparable, with 36.9±13.2% in siNeg cells and 37.6±14.8% in siFyn cells (Figure S4 *D*, framed). By contrast, siRNA-mediated knock-down of LAT (siLAT) (for LAT expression levels see Figure 4*C*) significantly impaired Ca^2+^ signaling for both stimuli. The number of cells showing Ca^2+^ release patterns in the cell population decreased from 89.4±10.1% in siNeg cells to 73.5±5.8% in siLAT cells upon anti-CD3 stimulation. Upon anti-CD59 stimulation the number of Ca^2+^ release patterns significantly decreased to 13.0±6.6% in siLAT cells compared to 36.9±13.2% in siNeg cells (Figure 4*B*, framed). In addition, Ca^2+^ signaling was essentially abolished in LAT-deficient J.CaM2.5 cells for both anti-CD3 and anti-CD59 stimulation (3.0±2.3% and 1.7±1.6% Ca^2+^ release patterns, respectively) (Figure 4*B*, framed), with CD3 and CD59 expression levels similar to that of WT cells (Figure S4 *B*). Altogether, these data demonstrated that Fyn is dispensable for anti-CD59 stimulation, whereas loss of Lck or LAT greatly impairs CD59-mediated signaling.

### Lck expression levels affect TCR/CD3 surface expression upon CD59- and TCR/CD3-mediated stimulation

**Figure 5:**
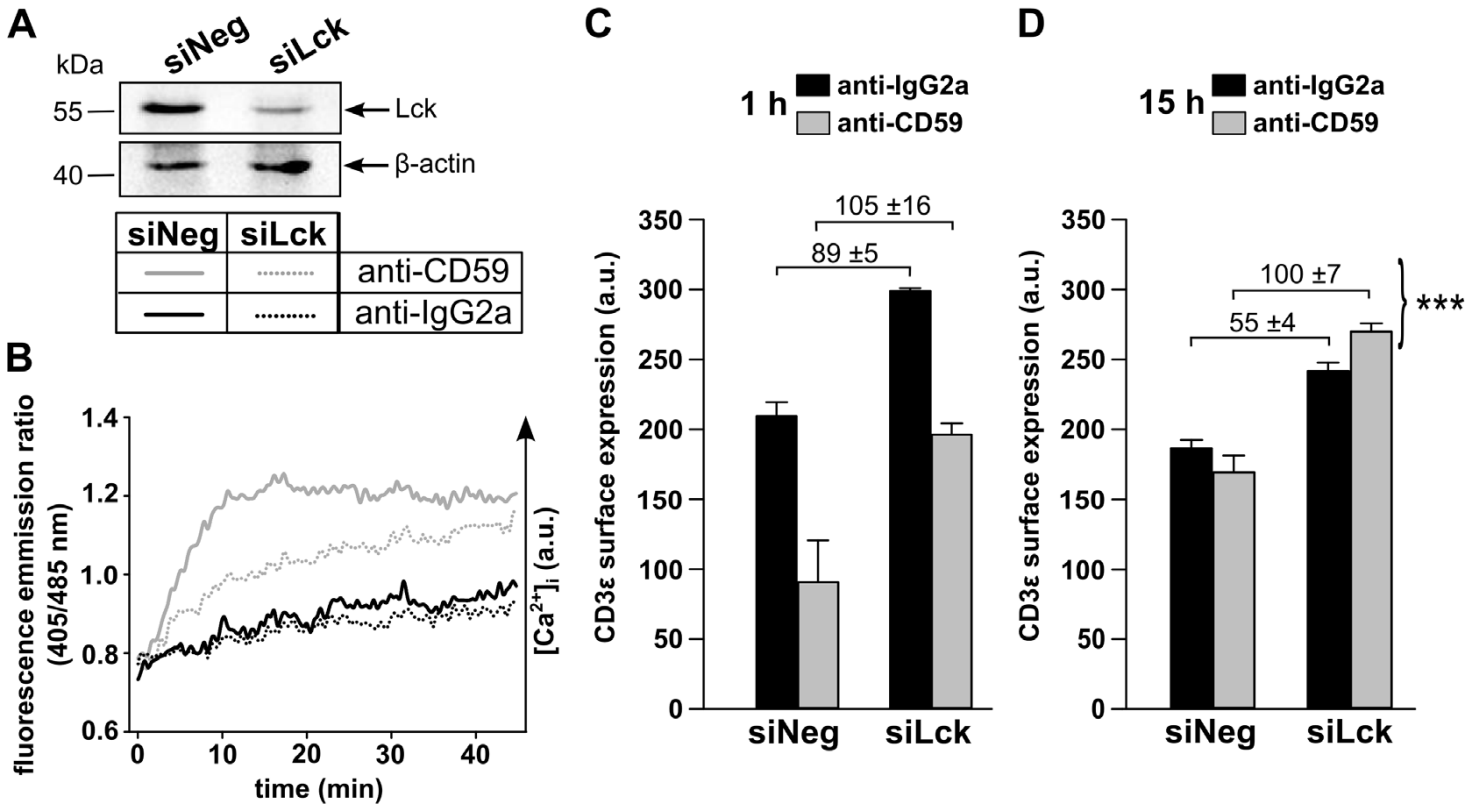
Lck expression and anti-CD59 stimulation influence CD3 surface expression. WT cells were transfected with negative control siRNA (siNeg) or Lck-specific siRNA (siLck) and experiments were performed 48 h after transfection. (A) Testing of Lck knock-down efficiency. Cell lysates were probed by Western blotting for Lck expression and β-actin as a control. (B) Efficiency of Lck knock-down tested by ensemble Ca^2+^ measurements. siLck and siNeg cells were loaded with Indo-1/AM. Cells were incubated with anti-CD59 mAb or anti-IgG2a for 5 min at 37 °C. For antibody cross-linking, goat anti-mouse F(ab’)_2_ was added to samples and Ca^2+^ mobilization of the whole cell population was measured by a microplate reader. For testing TCR surface expression levels, cells were stimulated with anti-CD59 or anti-IgG2a, as isotype control, followed by incubation with goat anti-mouse F(ab’)_2_ for (C) 1 h or (D) 15 h at 37°C. Cells were surface stained at 4 °C with FITC-conjugated anti-CD3ε or isotype control and analyzed by flow cytometry. Live cells were gated based on the Forward Scatter and Side Scatter profiles and propidium iodide exclusion. Fluorescence values displayed are isotype control corrected. Representative results of two separate experiments are shown (mean ±SD, n = 4). Multiple comparison tests were assessed by one-way ANOVA, significances are shown where applicable, ***p < 0.001.

In the knock-down experiments Lck was identified as the most upstream possible candidate molecule for linking the CD59- and TCR/CD3-mediated signaling pathways. Triggering of the TCR/CD3-mediated signaling pathway has been shown to down-modulate TCR/CD3 complex expression at the cell surface, a process which does not require Lck. However, Lck was found to be crucial for subsequent degradation of the internalized TCR/CD3 complex, as reflected in an enhanced recovery of TCR surface expression upon Lck knock-down or inhibition [32], [33]. Assuming that the CD59-mediated signaling pathway is coupled into the TCR/CD3-mediated signaling pathway, we tested if anti-CD59 stimulation triggers similar effects on CD3 surface expression as reported for direct TCR/CD3 stimulation. For this, we followed CD3ε surface expression upon anti-CD59 stimulation of Jurkat cells expressing normal and low levels of Lck (siNeg and siLck, respectively). Low Lck expression levels were achieved by siRNA-mediated protein knock-down, where total protein expression levels were tested by Western blotting (Figure 5*A*). The efficiency of reducing Lck levels was also tested in functional assays, measuring attenuated Ca^2+^ signaling upon anti-CD59 stimulation (Figure 5*B*).

Notably, CD3ε surface expression levels were *per se* up-regulated in siLck cells, which can be explained by reduced TCR/CD3 complex degradation in these cells [33]. Anti-CD59 stimulation caused a significant down-regulation of CD3ε surface expression, independent of Lck expression levels (Figure 5*C*). However, allowing degradation of the TCR/CD3 complex for 15 h after anti-CD59 stimulation pointed to a difference between siNeg and siLck cells. Although recovery of CD3ε surface expression could take place for both siNeg and siLck cells, recovery in siLck cells was significantly enhanced (Figure 5*D*). This indicated a role for Lck influencing the fate of the TCR/CD3 complex upon anti-CD59 stimulation similar to that upon anti-CD3 stimulation.

### Lck couples CD59-mediated signaling into the TCR/CD3-mediated signaling pathway

**Figure 6:**
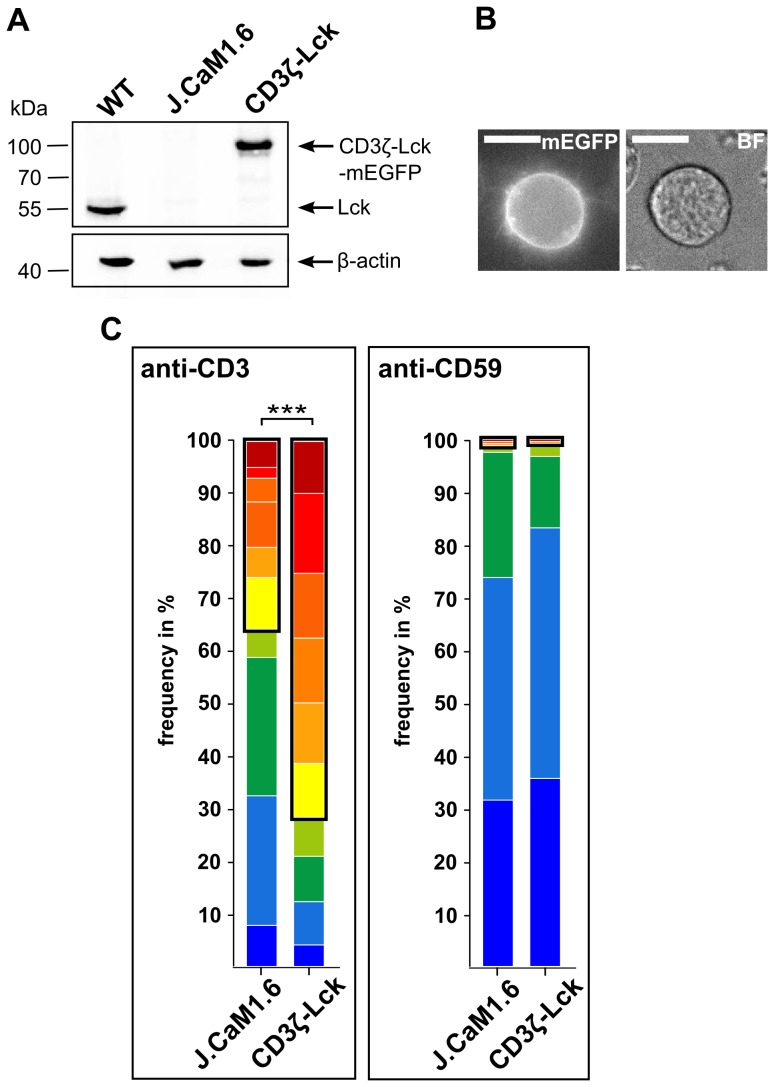
Reconstitution of Lck by forced interaction of CD3ζ and Lck facilitates TCR/CD3- but not CD59-mediated Ca^2+^ signaling. (A) Lck expression levels in WT cells, J.CaM1.6 cells and J.CaM1.6 cells expressing mEGFP-tagged Lck fused to CD3ζ (CD3ζ-Lck) were tested by Western blotting, the same blot was reprobed using anti-β-actin as a control. (B) Plasma membrane localization of CD3ζ-Lck-mEGFP in J.CaM1.6 cells was imaged by fluorescence microscopy. CD3ζ-Lck-mEGFP fluorescence and a bright field (BF) image of a transfected J.CaM1.6 cell are shown (scale bars  =  10 µm). (C) Cluster distribution of Ca^2+^ time traces in Lck-deficient J.CaM1.6 cells and J.CaM1.6 cells stably expressing mEGFP-tagged Lck fused to CD3ζ (CD3ζ-Lck) is shown upon anti-CD3 or anti-CD59 stimulation. Each color represents the percentage of a certain Ca^2+^ time trace cluster in the cell population. Clusters representing Ca^2+^ release patterns are framed in black (35.7±4.9% and 71.8±2.3% upon anti-CD3 stimulation, 1.4±2.3% and 1.0±1.3% upon anti-CD59 stimulation for J.CaM1.6 and CD3ζ-Lck cells, respectively). Mean values from three independent experiments, each with three technical replicates, are shown (n ≥ 323 per cell type and condition). Multiple comparison tests for the fractions showing Ca^2+^ release patterns were assessed by one-way ANOVA, significances are shown where applicable, ***p < 0.001.

Next we tested if CD59-mediated signaling can be triggered when Lck is physically associated with the TCR/CD3 machinery. For this, we designed a construct where the N-terminus of WT Lck is fused to the transmembrane domain of CD3ζ. Lck-deficient (J.CaM1.6) cells were transfected with the CD3ζ-Lck fusion construct, which reconstituted Lck expression to the WT level (Figure 6*A*). Tagging of the CD3ζ-Lck fusion protein C-terminally with mEGFP allowed the enrichment of CD3ζ-Lck-positive cells in the population and testing for membrane expression in each cell (Figure 6*B*). Single-cell Ca^2+^ measurements for untransfected J.CaM1.6 cells and CD3ζ-Lck-positive J.CaM1.6 cells (for clustering of single-cell Ca^2+^ time traces see Figure S5) revealed that the demonstrated Lck reconstitution significantly enhanced Ca^2+^ signaling upon anti-CD3 stimulation (35.7±4.9% vs. 71.8±2.3% Ca^2+^ release patterns, respectively) (Figure 6*C*, framed). However, neither untransfected J.CaM1.6 cells nor CD3ζ-Lck-positive J.CaM1.6 cells were responsive upon anti-CD59 stimulation (1.4±2.3% and 1.0±1.3% Ca^2+^ release patterns, respectively) (Figure 6*C*, framed). These data showed that Lck in a form bound to CD3ζ is fully functional upon direct TCR/CD3 triggering but not sufficient for retaining CD59-mediated Ca^2+^ signaling.

## Discussion

Accumulating evidence suggests that CD59 is involved in modulating the T cell response. It has been shown that stimulation of Jurkat cells through CD59 leads to protein phosphorylation, proliferation and cytokine release [8], [9]. Although similar findings have been reported for other GPI-anchored molecules, such as CD55 and CD73 [34], recent studies highlighted an antigen-specific inhibitory effect of CD59 on human T cell responses [11], [12]. Nonetheless, the mechanism through which engaged CD59 could influence T cell signaling remained unclear. Here we show in Jurkat T cells that signaling elicited via CD59 engagement is integrated into the TCR/CD3 signaling pathway, which is mediated at least partially through a mobile fraction of Lck.

Various Ca^2+^ responses have been observed in T cell populations [35]–[37], where type and extent of the Ca^2+^ response could be related to the extent of T cell activation [25]. When we stimulated with anti-CD3ε- or anti-CD59-coated surfaces, the Jurkat cell population showed heterogeneous Ca^2+^ responses. Although heterogeneity was detected also in CD3 surface expression levels, it did not affect the threshold for triggering TCR/CD3-mediated Ca^2+^ signaling. This may be attributed to a reported generally increased signaling capacity of the Jurkat cell line [38]. By contrast, experiments with TCR/CD3 deficient or overexpressing cells demonstrated that TCR/CD3 expression is a crucial factor in CD59 signaling. Moreover, reconstituting ITAM domains by sole expression of CD3ζ subunits without the ligand-binding components of the TCR/CD3 complex partly restored Ca^2+^ signaling upon anti-CD59 stimulation, and pointed to CD3ζ being required for CD59-mediated signaling. These findings suggested that the CD59-mediated signaling pathway is coupled into the TCR/CD3 signaling machinery at a very proximal step, utilizing the ITAM domains of the TCR/CD3 complex.

CD59 is considered to be physically separated from the TCR/CD3 complex in the plasma membrane [5]–[7] and has been demonstrated to associate with the Src family tyrosine kinases Lck and Fyn in specialized membrane compartments [39], [40]. It has been previously hypothesized that CD59 signaling takes place through Ab-mediated co-aggregation of CD59 with the TCR/CD3 complex, leading to tyrosine kinase-mediated ITAM phosphorylation [41]. Testing for such a co-aggregation with state-of-the-art imaging approaches, we could not visualize physical interaction between these molecules on a second to minute time scale. However, the possibility of more transient dynamic interactions between CD3 and CD59 may not be excluded.

Nevertheless, by testing downstream components with siRNA-mediated protein knock-downs we revealed that the Src family tyrosine kinase Lck and the adaptor molecule LAT are essential for CD59-mediated signaling, as also reported for TCR/CD3 signaling [18], [42]. Interestingly, we found that TCR/CD3 surface expression was down-regulated upon triggering both direct TCR/CD3 and CD59 signaling. Moreover, Lck down-modulation appeared to facilitate re-expression of surface CD3 after anti-CD59 stimulation, similar to that has been shown upon direct TCR/CD3 trigger [33]. Therefore the above findings further supported a link between CD59 and the TCR/CD3 signaling pathway and implicated Lck in signal transmission.

Localization of CD59 to specialized membrane platforms, which are enriched in signaling molecules like Lck, appears to be a prerequisite for the observed signaling capacity of CD59 [41]. Moreover, it has been demonstrated that Ab-mediated cross-linking of CD59 results in phosphorylation of Lck [8], [10], [43], also considered as one of the most proximal steps in TCR/CD3-mediated signaling [1], [29]. The fact that the TCR/CD3 complex and CD59 are considered physically separated [5]–[7] and did not appear to interact upon anti-CD59 stimulation assumed a dynamic molecule transmitting the signal between these pathways. Lck has been shown to be specifically targeted to CD59 through the integral membrane protein MAL [44] and anchored to the plasma membrane via palmitoylation [45]. Consistent with an expected reversible nature of the Lck membrane anchor, a cytosolic pool has also been described [46]–[50]. At the same time, Zimmermann and his colleagues estimated an average lifetime of ∼50 s for Lck in the plasma membrane. The authors concluded that this is sufficient time for Lck to be activated or modified, allowing transmission of signals over distinct platforms of the whole T cell membrane [50]. Covalently binding Lck to CD3 in our experiments made Lck unavailable to CD59 and completely abolished signaling through this molecule. As TCR/CD3-mediated signaling was still functional, it is therefore unlikely that CD59 signaling triggered via Ab-coated surfaces is mediated through co-clustering of CD59 and the TCR/CD3 complex in our Jurkat model. This experiment also showed that Lck immobilized to CD3, even if it is functional in the TCR/CD3 pathway, is not sufficient for the signal transmission upon CD59 trigger. At the same time, several data presented in this work highlight the necessity of Lck in CD59 signaling in a similar fashion as it is involved in the TCR/CD3 pathway. One remarkable difference seen in our experimental system (Fig. 6) is that Lck needs to be mobile for transmitting the signal from CD59 to TCR/CD3, possibly through interaction with its ITAM domains. Whether the signal transfer is enabled by a cytosolic, endosomal, or membrane fraction of Lck, remains to be further explored.

Uncovering the molecular mechanisms by which accessory molecules expressed on the T cell surface may influence T cell activation, is key for understanding the complex and heterogeneous nature of T cell responses. Here we revealed a link between CD59 and CD3-mediated signaling in Jurkat T cells, where Lck is a key factor of signal transmission from CD59 to the TCR/CD3 complex. It is proposed that CD59 may act via Lck to modulate T cell signaling.

## Supporting Information

Figure S1 **Ca^2+^ imaging in living Jurkat cells.** (A) Schematic drawing of Jurkat cell stimulation. Indo-1/AM loaded cells were flushed onto Ab-coated glass slides and time-lapse imaging was performed for 8 minutes at a frame rate of 30 frames/min. (B) Indo-1/AM was excited at 333 nm and fluorescence emission was measured at 405 nm for the Ca^2+^-bound and 485 nm for the Ca^2+^-free form of Indo-1/AM, respectively. The ratio of fluorescence emission intensities at 405 nm and 485 nm reflects changes in [Ca^2+^]_i_ for each individual cell [27]. Ca^2+^ time traces of ∼50 cells were recorded simultaneously. For analysis, Ca^2+^ time traces were followed for 200 s after identification of initial surface contact for each individual cell. Fluorescence and false-color emission ratio images of a representative experiment and a representative Ca^2+^ time trace are shown.
(TIF)

Figure S2 **Stimulatory capacity of antibodies for TCR/CD3- and CD59-mediated Ca^2+^ signaling.** (A) Glass slides were coated with increasing concentrations of FITC-conjugated anti-CD3 or anti-CD59 mAb. The amount of surface-bound Ab was measured by fluorescence microscopy. Fluorescence intensities (mean ±SD, n = 3) are shown. (B) Cluster distribution of Ca^2+^ time traces in WT cells upon stimulation with varying anti-CD3 or anti-CD59 concentrations on the glass substrate. Each color represents the percentage of a certain Ca^2+^ time trace cluster in the cell population. Clusters representing Ca^2+^ release patterns are framed in black. Mean results of three technical replicates are shown (n ≥ 86 per stimulatory condition).
(TIF)

Figure S3 **Characterization of WT, TCR^-^, and TCR^high^ cells.** (A) Individual Ca^2+^ time traces from single-cell measurements were grouped into 12 clusters by affinity propagation clustering as described in Materials and Methods. Each plot shows the respective Ca^2+^ time traces for a cluster, an exemplar trace for each cluster is shown in black. Clusters representing Ca^2+^ release patterns are framed in black. (B) CD3ζ surface expression level in WT, TCR^-^, and TCR^high^ cells tested by flow cytometry. Cells were surface stained with FITC-conjugated anti-CD3ε. Live cells were gated based on the Forward Scatter and Side Scatter profiles and propidium iodide exclusion. Fluorescence values displayed are isotype control corrected (mean ±SD, n = 4). Multiple comparison tests were assessed by one-way ANOVA, significances are shown where applicable, *** p < 0.001. (C) Total CD3ζ levels in WT and TCR^high^ cells tested by Western blotting. Cell lysates were probed for CD3ζ expression and the same blot was reprobed using Lck as a loading control. The 43 kDa band represents CD3ζ-EYFP, the 16 kDa represents the endogenous CD3ζ. (D) Total Lck and CD59 levels in WT, TCR^high^, and TCR^-^ cells tested by Western blotting. Cell lysates were probed for CD59 and Lck expression and for β-actin as a loading control. (E) Transfection efficiency of CD8-ζ in TCR^-^ cells tested by flow cytometry. Cells were transiently transfected with control vector (ctrl) or CD8-ζ expression vector, followed by surface staining with FITC-conjugated anti-CD8a. Live cells were gated based on the Forward Scatter and Side Scatter profiles and propidium iodide exclusion. Fluorescence values displayed are isotype control corrected (mean ±SD, n = 4). Multiple comparison tests were assessed by one-way ANOVA, significances are shown where applicable, *** p < 0.001.
(TIF)

Figure S4 **Fyn is not essential for TCR/CD3- and CD59-mediated Ca^2+^ signaling.** (A) Individual Ca^2+^ time traces from single-cell measurements were grouped into 11 clusters by affinity propagation clustering as described in Materials and Methods. Each plot shows the respective Ca^2+^ time traces for a cluster, an exemplar trace for each cluster is shown in black. Clusters representing Ca^2+^ release patterns are framed in black. (B) Total CD3ε and CD59 levels in WT, J.CaM1.6, and J.CaM2.5 cells tested by Western blotting. Cell lysates were probed for CD59 and Lck expression and the same blot was reprobed using GAPDH as a loading control. (C) Knock-down efficiency of Fyn was tested by Western blotting. 48 h after transfection, cell lysates from cells treated with Fyn-specific siRNA were probed with anti-Fyn and anti-β-actin as a control. (D) Cluster distribution of Ca^2+^ time traces in Jurkat cells transfected with negative control siRNA (siNeg) or Fyn-specific (siFyn) upon anti-CD3 or anti-CD59 stimulation. Each color represents the percentage of a certain Ca^2+^ time trace cluster in the cell population. Clusters representing Ca^2+^ release patterns are framed in black (89.4±10.1% and 79.6±11.0% upon anti-CD3 stimulation, 36.9±13.2% and 37.6±14.8% upon anti-CD59 stimulation for siNeg and siFyn cells, respectively). Mean values from five independent experiments, each with three technical replicates, are shown (n ≥ 288 per cell type and condition). Multiple comparison tests were assessed by one-way ANOVA.
(TIF)

Figure S5 **Reconstitution of Lck by forced interaction of CD3ζ and Lck facilitates TCR/CD3- but not CD59-mediated Ca^2+^ signaling.** Individual Ca^2+^ time traces from single-cell measurements were grouped into 10 clusters by affinity propagation clustering as described in Materials and Methods. Each plot shows the respective Ca^2+^ time traces for a cluster, an exemplar trace for each cluster is shown in black. Clusters representing Ca^2+^ release patterns are framed in black.
(TIF)
