# Supplementary figures and images for: Lck Mediates Signal Transmission from CD59 to the TCR/CD3 Pathway in Jurkat T Cells
Source: PLoS One. 2014 Jan 15;9(1):e85934. doi: 10.1371/journal.pone.0085934 (PMC3893272; doi:10.1371/journal.pone.0085934)

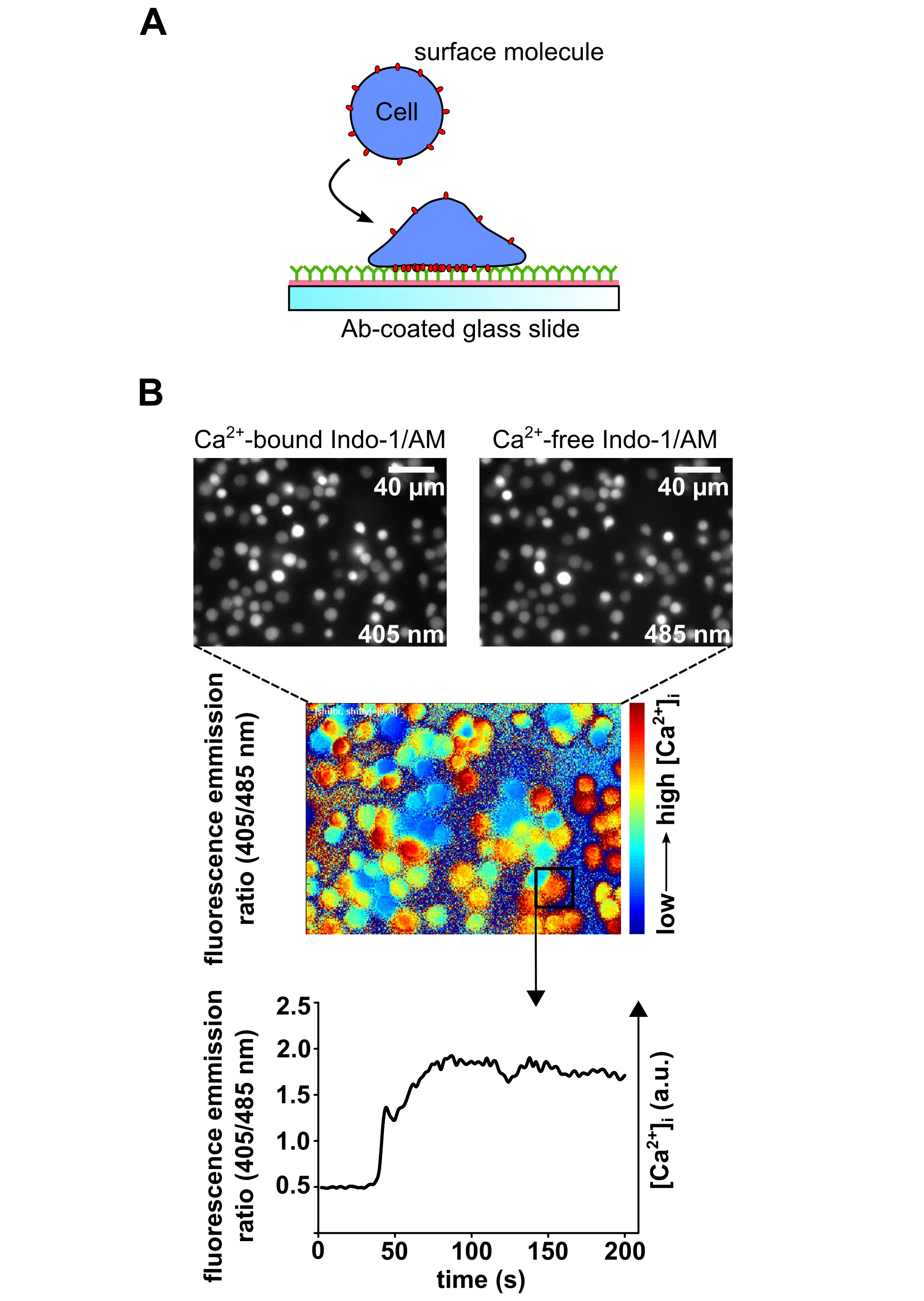

Supplement: Figure S1 — Ca2+ imaging in living Jurkat cells. (A) Schematic drawing of Jurkat cell stimulation. Indo-1/AM loaded cells were flushed onto Ab-coated glass slides and time-lapse imaging was performed for 8 minutes at a frame rate of 30 frames/min. (B) Indo-1/AM was excited at 333 nm and fluorescence emission was measured at 405 nm for the Ca2+-bound and 485 nm for the Ca2+-free form of Indo-1/AM, respectively. The ratio of fluorescence emission intensities at 405 nm and 485 nm reflects changes in [Ca2+]i for each individual cell [27]. Ca2+ time traces of ∼50 cells were recorded simultaneously. For analysis, Ca2+ time traces were followed for 200 s after identification of initial surface contact for each individual cell. Fluorescence and false-color emission ratio images of a representative experiment and a representative Ca2+ time trace are shown. (TIF) [file pone.0085934.s001.tif]

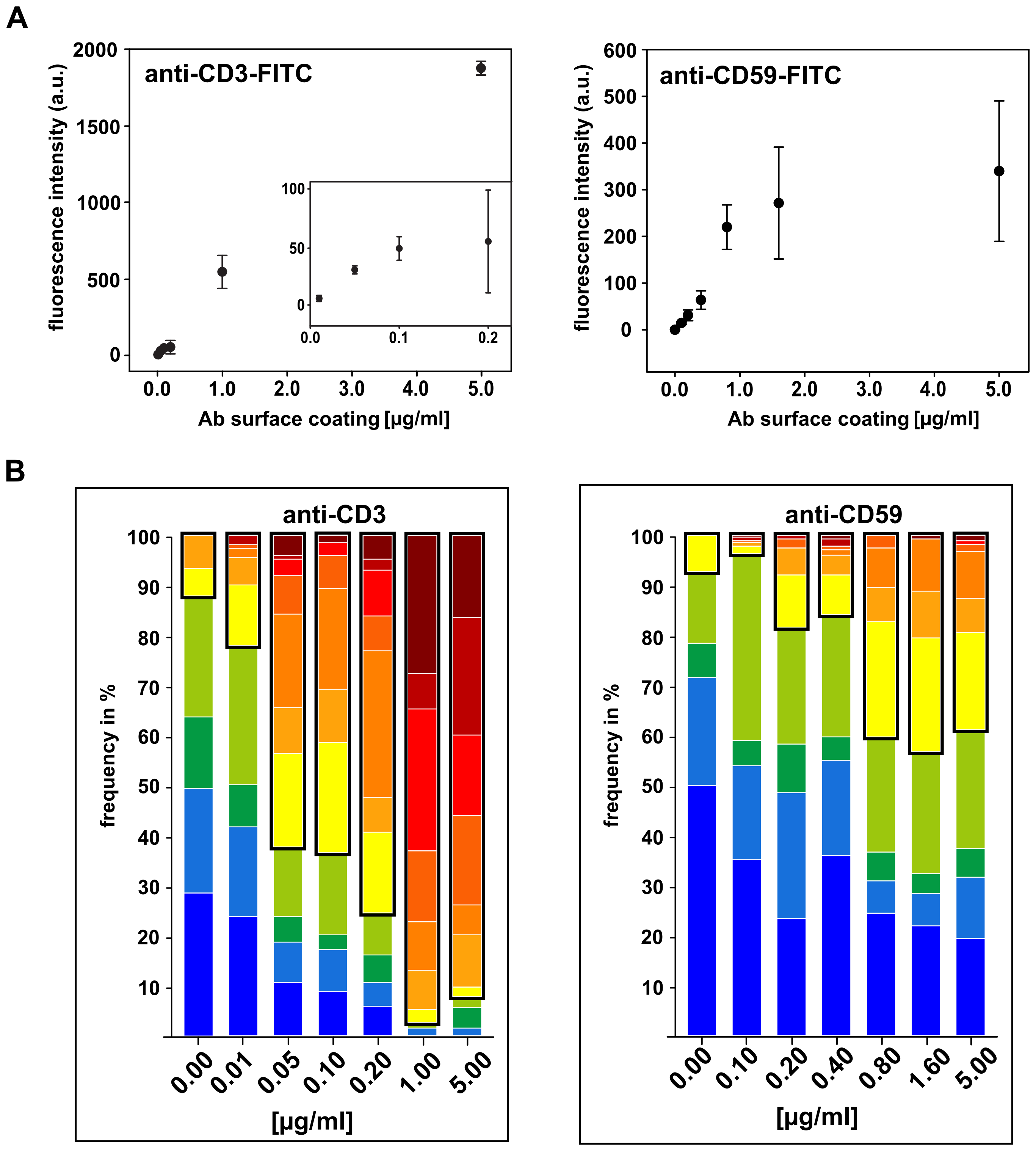

Supplement: Figure S2 — Stimulatory capacity of antibodies for TCR/CD3- and CD59-mediated Ca2+ signaling. (A) Glass slides were coated with increasing concentrations of FITC-conjugated anti-CD3 or anti-CD59 mAb. The amount of surface-bound Ab was measured by fluorescence microscopy. Fluorescence intensities (mean ±SD, n = 3) are shown. (B) Cluster distribution of Ca2+ time traces in WT cells upon stimulation with varying anti-CD3 or anti-CD59 concentrations on the glass substrate. Each color represents the percentage of a certain Ca2+ time trace cluster in the cell population. Clusters representing Ca2+ release patterns are framed in black. Mean results of three technical replicates are shown (n ≥ 86 per stimulatory condition). (TIF) [file pone.0085934.s002.tif]

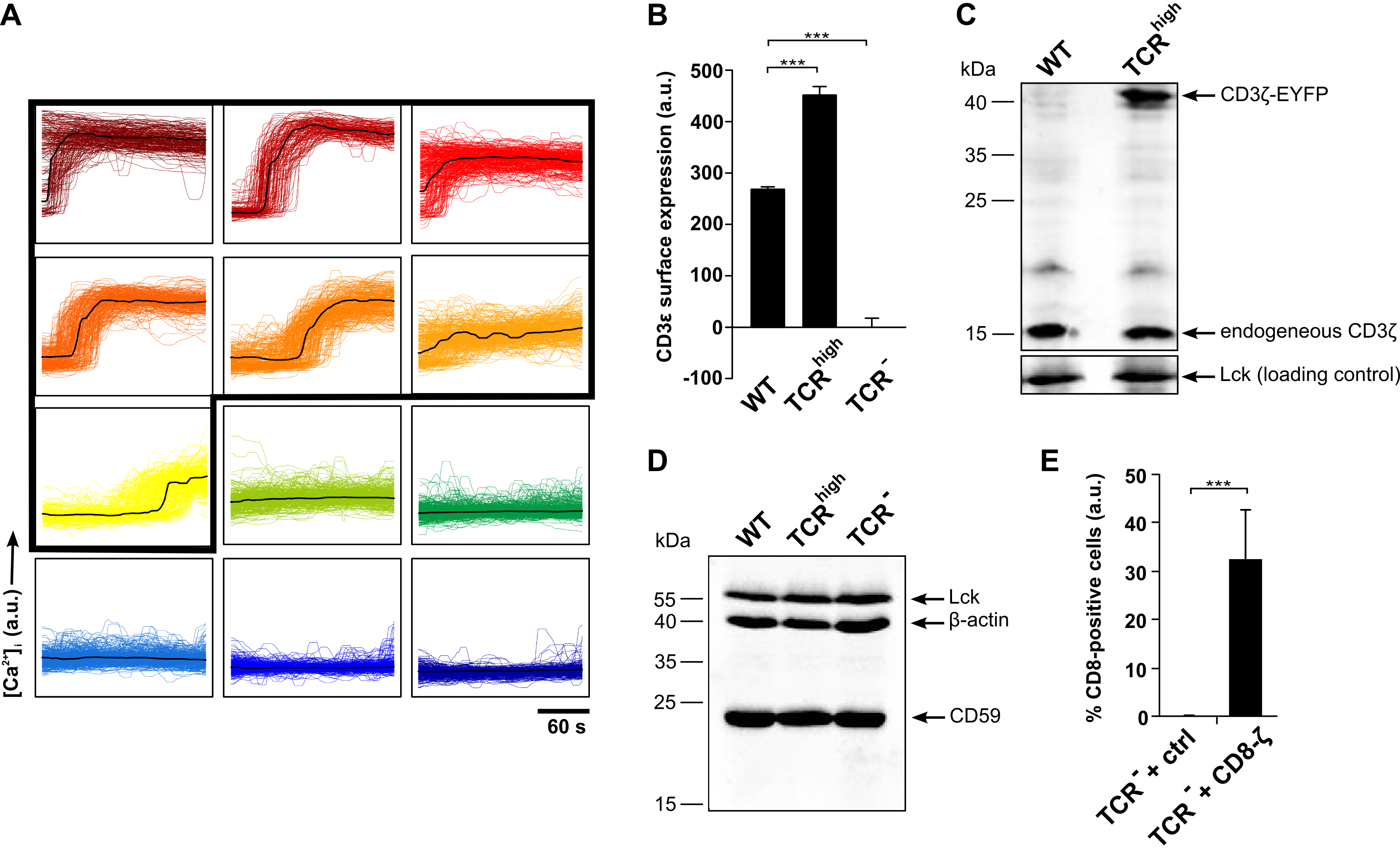

Supplement: Figure S3 — Characterization of WT, TCR-, and TCRhigh cells. (A) Individual Ca2+ time traces from single-cell measurements were grouped into 12 clusters by affinity propagation clustering as described in Materials and Methods. Each plot shows the respective Ca2+ time traces for a cluster, an exemplar trace for each cluster is shown in black. Clusters representing Ca2+ release patterns are framed in black. (B) CD3ζ surface expression level in WT, TCR-, and TCRhigh cells tested by flow cytometry. Cells were surface stained with FITC-conjugated anti-CD3ε. Live cells were gated based on the Forward Scatter and Side Scatter profiles and propidium iodide exclusion. Fluorescence values displayed are isotype control corrected (mean ±SD, n = 4). Multiple comparison tests were assessed by one-way ANOVA, significances are shown where applicable, *** p < 0.001. (C) Total CD3ζ levels in WT and TCRhigh cells tested by Western blotting. Cell lysates were probed for CD3ζ expression and the same blot was reprobed using Lck as a loading control. The 43 kDa band represents CD3ζ-EYFP, the 16 kDa represents the endogenous CD3ζ. (D) Total Lck and CD59 levels in WT, TCRhigh, and TCR- cells tested by Western blotting. Cell lysates were probed for CD59 and Lck expression and for β-actin as a loading control. (E) Transfection efficiency of CD8-ζ in TCR- cells tested by flow cytometry. Cells were transiently transfected with control vector (ctrl) or CD8-ζ expression vector, followed by surface staining with FITC-conjugated anti-CD8a. Live cells were gated based on the Forward Scatter and Side Scatter profiles and propidium iodide exclusion. Fluorescence values displayed are isotype control corrected (mean ±SD, n = 4). Multiple comparison tests were assessed by one-way ANOVA, significances are shown where applicable, *** p < 0.001. (TIF) [file pone.0085934.s003.tif]

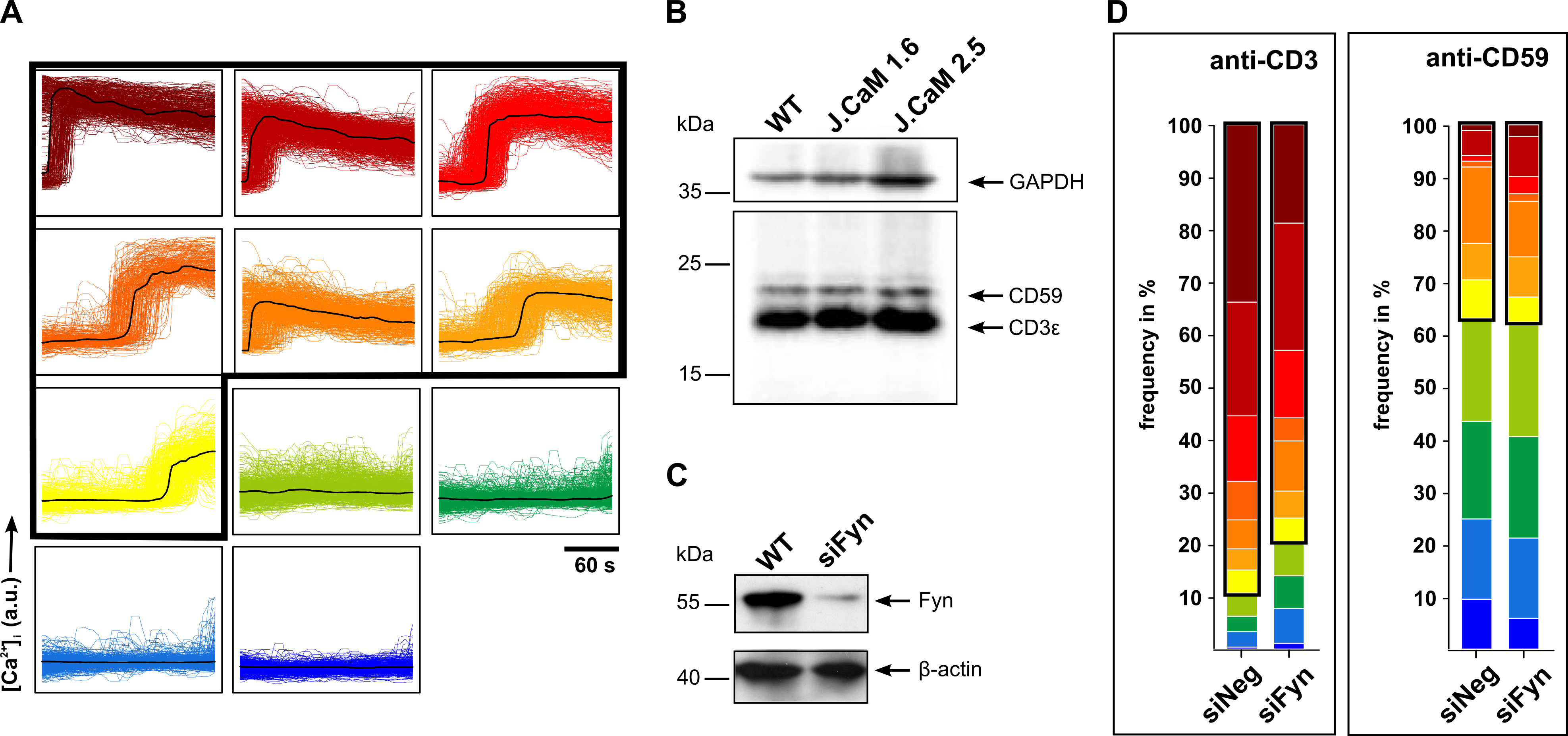

Supplement: Figure S4 — Fyn is not essential for TCR/CD3- and CD59-mediated Ca2+ signaling. (A) Individual Ca2+ time traces from single-cell measurements were grouped into 11 clusters by affinity propagation clustering as described in Materials and Methods. Each plot shows the respective Ca2+ time traces for a cluster, an exemplar trace for each cluster is shown in black. Clusters representing Ca2+ release patterns are framed in black. (B) Total CD3ε and CD59 levels in WT, J.CaM1.6, and J.CaM2.5 cells tested by Western blotting. Cell lysates were probed for CD59 and Lck expression and the same blot was reprobed using GAPDH as a loading control. (C) Knock-down efficiency of Fyn was tested by Western blotting. 48 h after transfection, cell lysates from cells treated with Fyn-specific siRNA were probed with anti-Fyn and anti-β-actin as a control. (D) Cluster distribution of Ca2+ time traces in Jurkat cells transfected with negative control siRNA (siNeg) or Fyn-specific (siFyn) upon anti-CD3 or anti-CD59 stimulation. Each color represents the percentage of a certain Ca2+ time trace cluster in the cell population. Clusters representing Ca2+ release patterns are framed in black (89.4±10.1% and 79.6±11.0% upon anti-CD3 stimulation, 36.9±13.2% and 37.6±14.8% upon anti-CD59 stimulation for siNeg and siFyn cells, respectively). Mean values from five independent experiments, each with three technical replicates, are shown (n ≥ 288 per cell type and condition). Multiple comparison tests were assessed by one-way ANOVA. (TIF) [file pone.0085934.s004.tif]

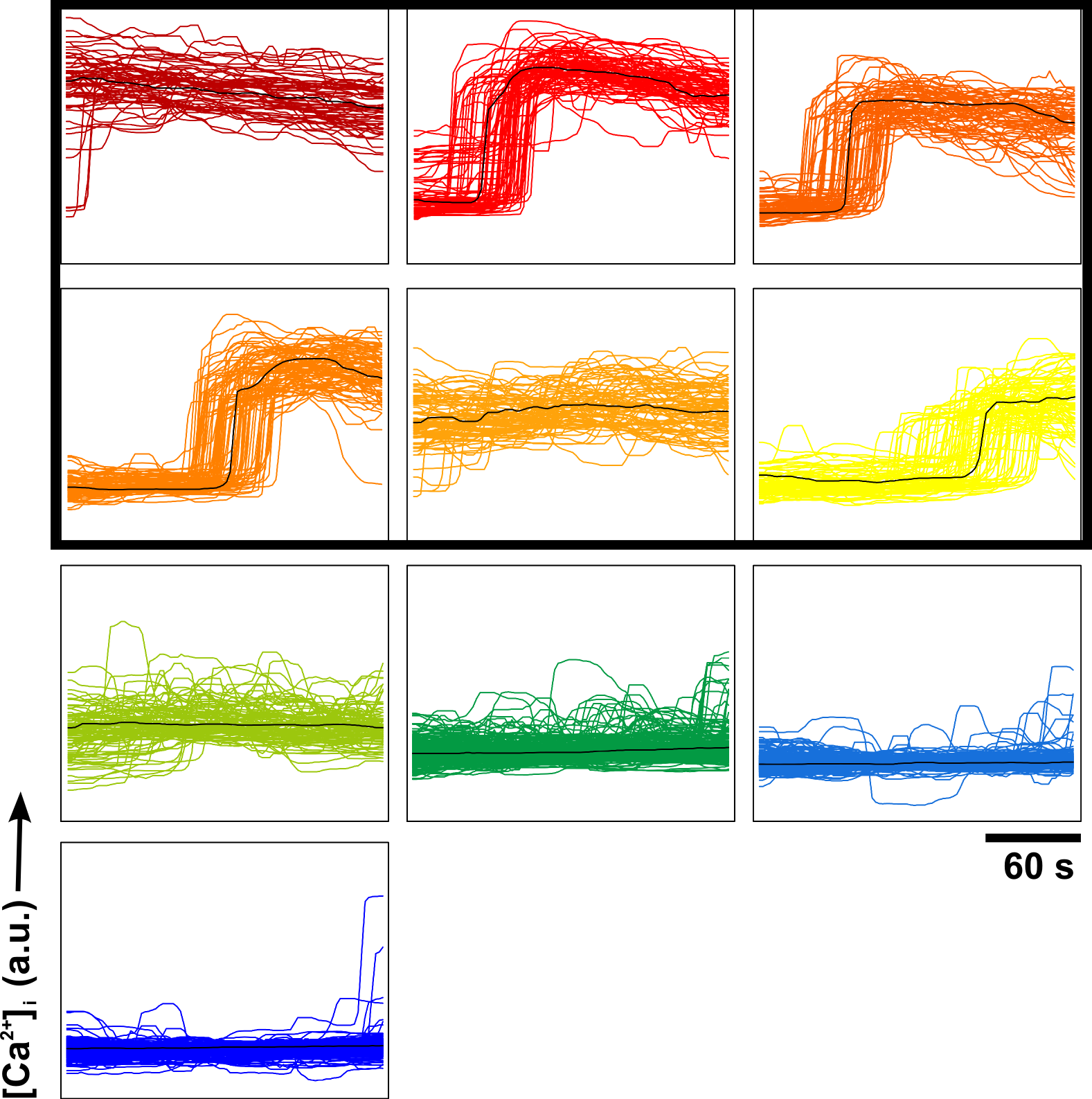

Supplement: Figure S5 — Reconstitution of Lck by forced interaction of CD3ζ and Lck facilitates TCR/CD3- but not CD59-mediated Ca2+ signaling. Individual Ca2+ time traces from single-cell measurements were grouped into 10 clusters by affinity propagation clustering as described in Materials and Methods. Each plot shows the respective Ca2+ time traces for a cluster, an exemplar trace for each cluster is shown in black. Clusters representing Ca2+ release patterns are framed in black. (TIF) [file pone.0085934.s005.tif]
